# Polyhistidine facilitates direct membrane translocation of cell-penetrating peptides into cells

**DOI:** 10.1038/s41598-019-45830-8

**Published:** 2019-06-28

**Authors:** Han-Jung Lee, Yue-Wern Huang, Shiow-Her Chiou, Robert S. Aronstam

**Affiliations:** 1 Department of Natural Resources and Environmental Studies, National Dong Hwa University, Hualien, 974 Taiwan; 2 Department of Biological Sciences, Missouri University of Science and Technology, Rolla, MO 65409-1120 USA; 3 Graduate Institute of Microbiology and Public Health, National Chung Hsing University, Taichung, 402 Taiwan; 4 College of Science and Technology, Bloomsburg University of Pennsylvania, Bloomsburg, PA 17815-1301 USA

**Keywords:** Drug delivery, Cell delivery

## Abstract

The bovine lactoferricin L6 (RRWQWR) has been previously identified as a novel cell-penetrating peptide (CPP) that is able to efficiently internalize into human cells. L6 interacts with quantum dots (QDs) noncovalently to generate stable L6/QD complexes that enter cells by endocytosis. In this study, we demonstrate a modified L6 (HL6; CHHHHHRRWQWRHHHHHC), in which short polyhistidine peptides are introduced into both flanks of L6, has enhanced cell-penetrating ability in human bronchoalveolar carcinoma A549 cells. The mechanism of cellular uptake of HL6/QD complexes is primarily direct membrane translocation rather than endocytosis. Dimethyl sulfoxide (DMSO), but not pyrenebutyrate (PB), ethanol, oleic acid, or 1,2-benzisothiazol-3(2 *H*)-one (BIT), slightly enhances HL6-mediated protein transduction efficiency. Neither HL6 nor HL6/QD complexes are cytotoxic to A549 or HeLa cells. These results indicate that HL6 could be a more efficient drug carrier than L6 for biomedical as well as biotechnological applications, and that the function of polyhistidine peptides is critical to CPP-mediated protein transduction.

## Introduction

Cell-penetrating peptides (CPPs) are now widely used as a highly effective strategy for delivery of bioactive macromolecules into cells using protein transduction^1^. CPPs, also called peptide/protein transduction domains (PTDs), comprise a family of short peptides, typically made of 5–30 amino acids, that can pass through tissues and cell membranes, and internalize into cells without affecting cell viability^2,3^. Under physiological conditions, CPPs generally have positive charges and/or are amphipathic. This allows them to be capable of binding to negatively charged glycosaminoglycans (GAGs)^4^ and recruit class A scavenger receptors^5^ on the surface of cell membranes. CPPs are subsequently internalized into the cytoplasm primarily by either endocytosis or direct membrane translocation (direct penetration)^2,6^. Importantly, CPPs can carry a variety of biologically functional molecules, including DNAs, RNAs, proteins, small drugs, and nanomaterials, into cells. These cargos can be conjugated to CPPs using covalent bonds or noncovalent complex formation^2,6^. Disulfide and thioester bonds can be used to covalently couple CPPs to cargos (denoted as CPP-cargo). Molecular cloning and the subsequent expression of CPP-fused proteins is another effective way to form covalent couples in CPPs capable of binding to cargo. In contrast, noncovalent complex formation relies on electrostatic interactions shared by both positively charged CPPs and negatively charged cargos (denoted as CPP/cargo) or by hydrophobic interactions^2^.

CPPs are able to internalize into cells by either endocytosis or direct membrane translocation^6^. Most CPPs and CPP/cargo complexes rely on endocytosis to be incorporated into cells^7^. Endocytosis is an energy-dependent process that occurs in all living cells. Endocytosis encompasses several different pathways that can be divided into an actin-dependent macropinocytosis, a clathrin- or caveolae-dependent pathway, and a clathrin/caveolae-independent route^8^. Vesicle content usually enters the endo-lysosomal system where it is eventually digested in acid hydrolases-containing lysosomes^9^. Formation of endosomal vesicles in endocytosis requires at least 5–15 min^10,11^. In CPP-mediated delivery of functional macromolecules, endosomal escape into the cytoplasm by endocytic pathway may be the most important rate-limiting factor^3^. Direct membrane translocation is a rapid and energy-independent process utilized by some CPPs, such as HR9^12^, which make the plasma membrane unstable, thereby producing transient pores for cellular penetration^13^. Direct membrane translocation may involve multiple mechanisms, as exemplified by the carpet-like and inverted-micelle models that are related to transient pore formation or membrane destabilization^2^. The direct membrane translocation pathway may be a more effective route to convey CPP-derived pharmaceutics and therapeutics, insofar as it does not entail endosomal entrapment^14^.

Administration concentrations of arginine-rich CPPs employed during protein transduction played a vital part in identifying two internalization pathways employed by CPPs^15^. For instance, it was found in one study that endocytosis is the main pathway at low CPP concentrations, while direct membrane translocation is noticeable at higher CPP concentrations than the threshold of 5 μM^15^. Arginine-rich CPPs dynamically alter local membrane structure during direct membrane translocation^15^, which originates from nucleation zones (NZs)^16^. These spatially restricted NZ regions on the plasma membrane provide potent platforms for CPP internalization^16^. Enzymatic digestion with heparinases strongly suppresses direct membrane translocation of arginine-rich CPPs, indicating a role of heparan sulfates on cell surface in internalization through NZs^15,16^.

In our previous study, a bovine lactoferricin L6 CPP^17^ has been demonstrated to noncovalently interact with carboxyl-functionalized quantum dots (QDs) and form stable L6/QD complexes that can enter cells by endocytosis^18^. The aim of the present study was to assess whether modification of L6 with additional penta-histidine and mono-cysteine residues on both sides would influence its 1) ability to form noncovalent complexes with QDs, 2) transduction efficiency, and 3) mechanism of cellular uptake.

## Materials and Methods

### Cell culture

Human bronchoalveolar carcinoma A549 cells (American Type Culture Collection, Manassas, VA, USA; CCL-185) were grown in Gibco Roswell Park Memorial Institute (RPMI) 1640 medium (Thermo Fisher, Waltham, MA, USA) supplemented with 10% (v/v) fetal bovine serum (FBS, Thermo Fisher), 1× antibiotic-antimycotic solution (Caisson Labs, Smithfield, UT, USA), and 0.5 μl/ml of Cellmaxin Plus (GenDEPOT, Katy, TX, USA). Human cervical carcinoma HeLa cells (ATCC; CCL-2) were maintained in Eagle’s Minimum Essential Medium (MEM, Thermo Fisher) supplemented with 10% (v/v) FBS, 1× antibiotic-antimycotic solution, and 0.5 μl/ml of Cellmaxin Plus. All cells were cultured at 37 °C in a humidified incubator with 5% CO_2_.

### Preparation of peptides and QDs

HL6 (CHHHHHRRWQWRHHHHHC), N-terminal fluorescein isothiocyanate (FITC) labeled HL6 (FITC-HL6), FITC-L6 (RRWQWR)^17^, FITC-HR9 (CHHHHHRRRRRRRRRHHHHHC)^12^, and FITC-nonCPP (RPPGFSPFR, bradykinin)^19^ peptides were purchased from Genomics (Taipei, Taiwan). Carboxyl-functionalized and water-soluble QDs (CdSe/ZnS-PEG-COOH-520) with a maximal emission wavelength of 523 nm were purchased from Mesolight (Zhejiang, China).

### Gel retardation assay

To characterize noncovalent interactions between CPPs and QDs, a series of concentrations of HL6 ranging from 0 to 2.5 μM were incubated with 0.1 μM of QDs at 37 °C for 1 h. Complexes formed at molar ratios of 0 (QDs alone), 5, 10, 15, 20, and 25 were analyzed by electrophoresis on a 1% SeaKem Gold agarose gel (Lonza Group, Basel, Switzerland) in 0.5× TBE buffer (44.5 mM of Tris-borate and 1 mM of EDTA, pH 8.3) at 50 V for 30 min^20^. ChemiDoc XRS+ gel imaging system (Bio-Rad, Hercules, CA, USA) was used to capture and analyze gel images^17^. The relevant shift-percentage of a QD band shift in the gel is defined as a reciprocal mobility ratio normalized to scale in which the minimal molar ratio of 0 was 0%, and the maximal ratio of 25 was 100%.

### Noncovalent protein transduction

A549 cells were washed with phosphate buffered saline (PBS) and changed to serum-free RPMI 1640 medium for protein transduction experiments^21^. The presence of 10% serum in the medium as the solvent caused a decrease in the uptake of CPPs or their complexes/conjugates. For cellular internalization, cells were seeded at a density of about 1 × 10^5^ per 35-mm petri dish and then treated with serum-free medium (as a control), 80 μM of FITC isomer I (Sigma-Aldrich, St. Louis, MO, USA), FITC-nonCPP, FITC-L6, FITC-HL6, or FITC-HR9 at 37 °C for either 1 h or various time durations. The fluorescent tracker Hoechst 33342 was used to stain the nuclei of cells in blue, according to the manufacturer’s instructions (Thermo Fisher).

To analyze protein transduction of noncovalent CPP/QD complexes, 6 μM of HL6 peptides were premixed with 0.3 μM of QDs at 37 °C for 1 h. After complex formation, the mixtures were incubated with A549 cells at 37 °C for various durations^21^. Cells were washed with PBS to remove non-transduced material from cell surface.

### Fluorescent microscopy

Bright-field and fluorescent images were captured using an AE31 inverted Epi-fluorescence microscope (Motic, Hong Kong, China) equipped with an IS1000 eyepiece (Tucsen, Fujian, China)^17^. Excitation filter modules of green and blue fluorescent protein (GFP, BFP) channels were set at 480/30 and 350/50 nm, respectively. Emission filter modules of GFP and BFP channels were set at 535/40 and 460/50 nm, respectively.

### Flow cytometric analysis

Cells were seeded at a density of about 2 × 10^5^ per well in a 24-well plate. Following the protein transduction, cells were washed with PBS and harvested by adding 250 μl/well of 2.9 mM ethylenediaminetetraacetic acid (EDTA, pH 6.14)^22^. Samples were analyzed using a Cell Lab Quanta SC MPL flow cytometer (Beckman Coulter, Fullerton, CA, USA) possessed with 488 nm laser at a speed of 20 μl/min. Digital measurements were analyzed using Cell Lab Quanta SC MPL software version 1.0 (Beckman Coulter). Results are shown as the percentage of either the total cell population displaying green fluorescence or internalization efficiency.

### Mechanistic studies of cellular uptake

To determine potential cellular uptake pathways, low temperature (4 °C), various heparinases, and a series of pharmacological modulators [5-(*N*-ethyl-*N*-isopropyl)-amiloride (EIPA), cytochalasin D (CytD), filipin III (FIL), and nocodazole (NCO)] were applied (all inhibitors were purchased from Sigma-Aldrich). Low temperature (4 °C) treatment disrupts all energy-dependent movement across the cell membrane, and EIPA specifically arrests macropinocytosis^23^. CytD is an F-actin polymerization blocker that inhibits endocytic processes involving clathrin-, caveolae-dependent endocytosis, and macropinocytosis^24^. FIL inhibits lipid raft- and caveolae-dependent endocytosis^25^. NCO inhibits clathrin-dependent endocytosis^26^. Cells were pretreated at 4 °C for 30 min, and then treated with HL6/QD complexes (prepared at a molar ratio of 20 at 37 °C for 1 h) at 4 °C for an additional 5 or 30 min^27^. Cells were treated with HL6/QD complexes (prepared at a ratio of 20 at 37 °C for 1 h) in the presence or absence of 100 μM EIPA, 10 μM CytD, 5 μg/ml FIL, or 10 μM NCO at 37 °C for 5 or 30 min^27^. Cells were pretreated with heparinases I, II, and III (Sigma-Aldrich) from *Flavobacterium heparinum* (10, 5, and 2 U/ml, respectively) at 37 °C for 6 h, treated with HL6/QD complexes (prepared at a ratio of 20 at 37 °C for 1 h) at 37 °C for an additional 5 or 30 min^16^, and then analyzed using flow cytometry.

To assess the influence of chemical enhancers on protein transduction efficiency, cells were pretreated with or without 50 μM pyrenebutyrate (PB) at 37 °C for 2 min, 10% dimethyl sulfoxide (DMSO), 1% ethanol (EtOH), or 0.65 mM 1,2-benzisothiazol-3(2 *H*)-one (BIT) (Sigma-Aldrich) at 37 °C for 1 h. Subsequently, cells were treated with HL6/QD complexes (prepared at a ratio of 20 at 37 °C for 1 h) in the absence or presence of 50 μM PB, 10% DMSO, 1% EtOH, 80 μM oleic acid (OA), or 0.65 mM BIT at 37 °C for an additional 5 min^12,28^, and then analyzed using flow cytometry.

### Cytotoxicity assay

To assess the cytotoxicity of HL6, QDs, and HL6/QD complexes, both human A549 and HeLa cells were treated at 37 °C for 30 min with 6 μM of HL6 alone, 0.3 μM of QDs alone, or HL6/QD complexes (prepared at a ratio of 20 at 37 °C for 1 h). One hundred % DMSO and serum-free medium were used to treat cells for 30 min as positive and negative controls, respectively. HeLa cells were pretreated with 4 °C for 30 min or heparinases I, II, and III for 6 h. Cells were then treated with HL6/QD complexes (prepared at a ratio of 20 at 37 °C for 1 h) in the absence or presence of various endocytic inhibitors at 37 °C for 30 min. After treatment, cells were washed with PBS and then incubated in complete culture medium at 37 °C for 24 h. Eighty μl of serum-free medium and 20 μl of 3-(4,5-dimethylthiazol-2-yl)-2,5-diphenyltetrazolium bromide (MTT, Sigma-Aldrich; 5 mg/ml in PBS) solution were added to each well. After incubation at 37 °C for 30 min, soluble formazan crystals converted only by metabolically living cells were dissolved in 300 μl of DMSO. The 24-well plates were then read using a SpectraMax M2 microplate reader with SoftMax Pro 6.3 microplate analysis software (Molecular Devices, Sunnyvale, CA, USA) at 540 nm wavelength.

### Statistical analysis

Data are shown as mean ± standard deviation (SD) from more than three independent experiments performed in triplicates for each treatment group. Statistical comparisons were carried out by One-way ANOVA, using SigmaPlot software version 12.5 (Systat Software, San Jose, CA, USA) at levels of statistical significance when the *P*-value was less than 0.05 (*, α) or 0.01 (**, αα).

## Results

### Cellular internalization of HL6

**Figure 1:**
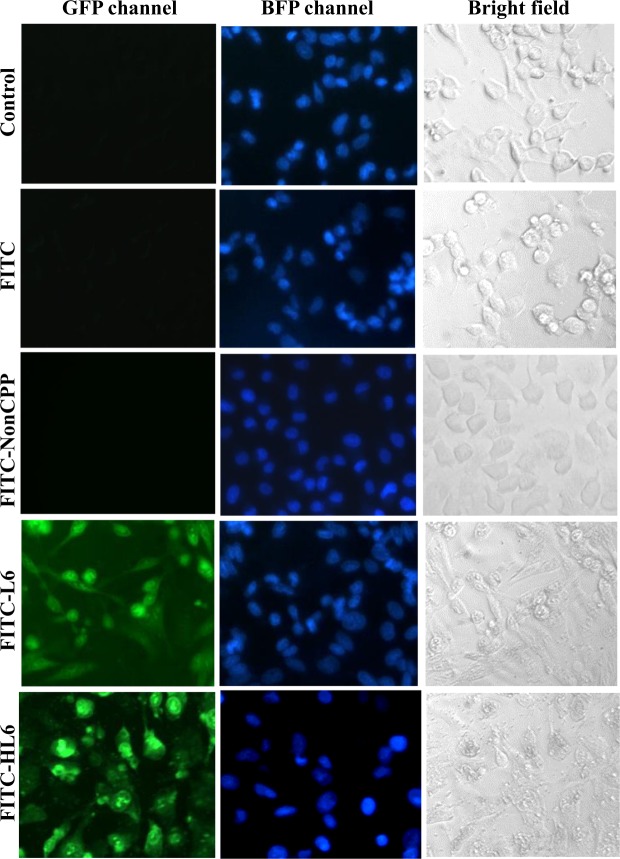
Cellular internalization of FITC-CPPs. Serum-free medium, FITC, and FITC-nonCPP served as controls were used to treat human A549 cells. Cells were treated with FITC-L6 and FITC-HL6 at 37 °C for 1 h, and subsequently stained with Hoechst 33342. Two fluorescent channels GFP and BFP disclosed cellular locations of FITC-CPP and nuclei, respectively. Bright-field images were obtained to represent cell morphologies. All images were observed and captured using a Motic AE31 fluorescent microscope with an enlargement of 200x.

HL6 internalization by human A549 cells was characterized by treating cells with FITC-CPPs and other vehicles at 37 °C for 1 h, followed by staining with Hoechst 33342. Fluorescent microscopy demonstrates green fluorescence in the cells treated with either FITC-L6 or FITC-HL6 (Fig. 1). Cells treated with serum-free medium, FITC, and FITC-nonCPP did not display green fluorescence, indicating that HL6 when comprised of L6 and polyhistidine is an effective CPP.

### A time course analysis of cellular internalization of HL6

**Figure 2:**
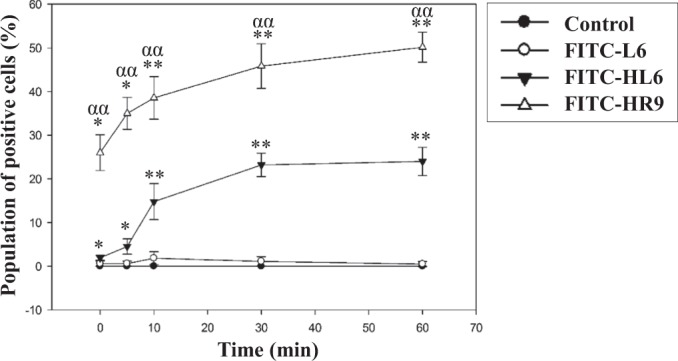
A time course of cellular internalization of FITC-CPPs. Serum-free medium, FITC-L6, FITC-HL6, and FITC-HR9 were used to treat A549 cells for different periods of time. Intracellularly fluorescent intensity was assayed by flow cytometric analysis. Significant differences were determined when the *P*-value was less than 0.05 (*, α) or 0.01 (**, αα). Data are shown as mean ± SD from seven independent experiments performed in triplicates for each treatment group.

The kinetics of HL6 internalization were evaluated using cells treated with FITC-HL6, FITC-L6 as an endocytic control (unpublished observations), or FITC-HR9 as a direct translocation control^12^ for various time durations, and then analyzed using a flow cytometer. FITC-L6 and control groups showed little green fluorescence at 0 min (Fig. 2). Green fluorescence was present in FITC-HL6 and FITC-HR9 groups within 5 min. Cellular internalization of FITC-HL6 and FITC-HR9 was consistently higher than FITC-L6 or control. At 60 min, uptake in the FITC-HL6 group was 46.7 times higher than in the FITC-L6 group. The fraction of cells showing uptake was higher with FITC-HR9-treated cells than FITC-HL6 and FITC-L6 at all time points. Endocytosis involves formation of endosomes that requires at least 5–15 min to form^10,11^, while HR9 has been proven to enter cells by direct membrane translocation^12^. Collectively, these results indicate that HL6 enters cells by direct membrane translocation just as HR9, but with a relatively lower efficiency.

### HL6 CPPs and QDs *in vitro*

**Figure 3:**
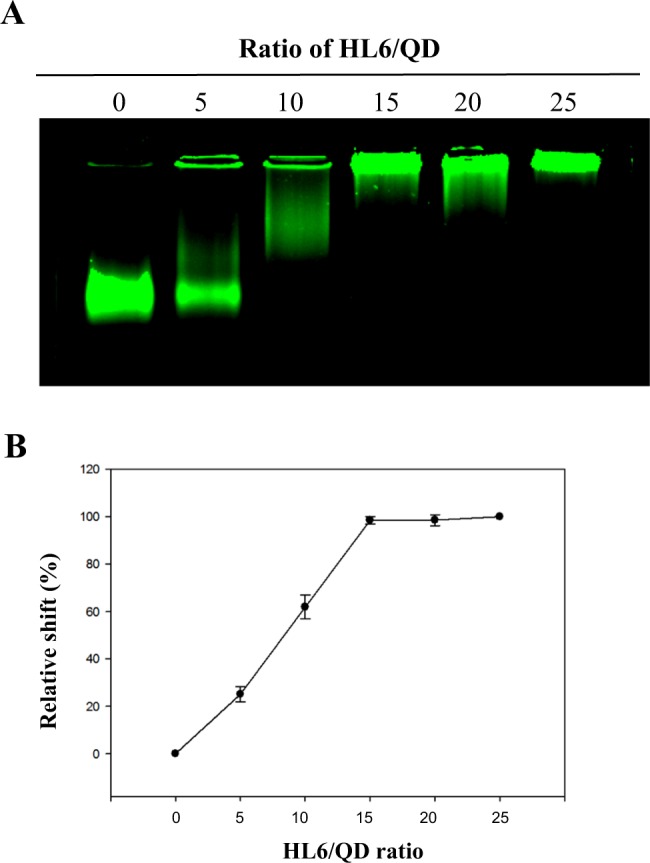
Noncovalent interactions between HL6 and QDs. (**A**) Gel retardation assay. Numerous amounts of HL6 were incubated with QDs at various molar ratios of 0 (QDs alone), 15, 30, 45, 60, and 75. HL6/QD complexes were analyzed by agarose-based gel electrophoresis, and a ChemiDoc XRS+ gel imaging system (Bio-Rad) was used to acquire fluorescent images. (**B**) The relative shift of HL6/QD complexes prepared at different molar ratios is indicated. Data were analyzed using the Quantity One 1-D analysis software version 4.6.9 (Bio-Rad) and presented as mean ± SD from five independent experiments for each treatment group.

The gel retardation assay was performed to determine whether HL6 CPPs form stable, noncovalent complexes with QDs. HL6 and QDs were incubated at molar ratios varying from 0 to 25. QDs mobility was found to be inversely related to the concentration of HL6 being incubated (Fig. 3A, Supplementary Fig. S1). Using relative shift analysis, ratio-dependent interactions of HL6/QD complexes were maximized at ratios above 15 (Fig. 3B). Accordingly, a molar ratio of 20 was selected and applied in subsequent experiments.

### Intracellular delivery of QDs using CPPs

**Figure 4:**
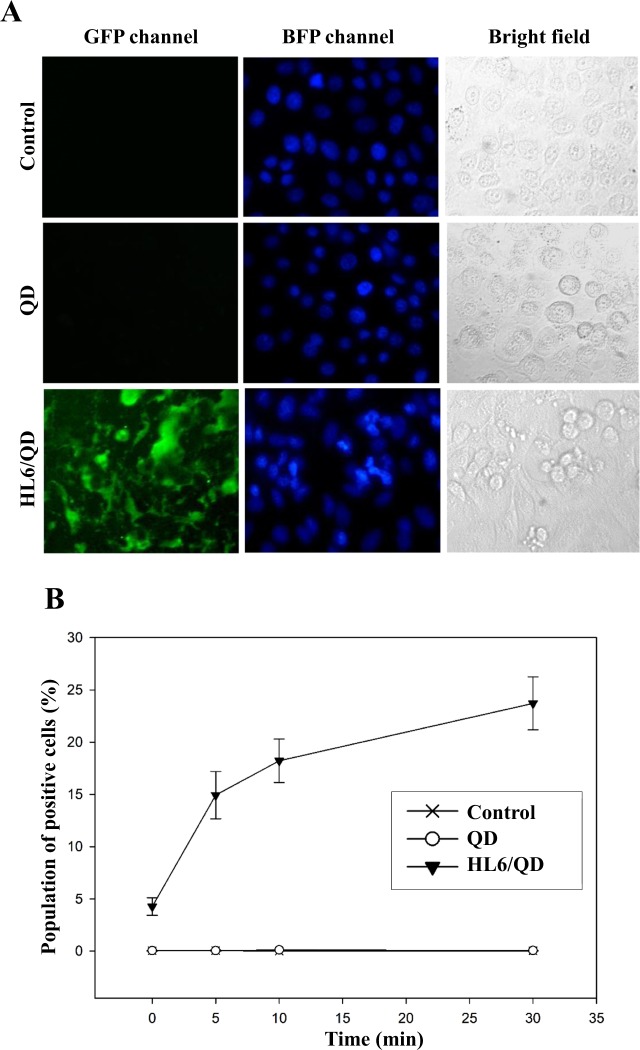
HL6-mediated cellular internalization of QDs. (**A**) Images of HL6-directed delivery of QDs into cells. A549 cells were treated with QDs alone or HL6/QD complexes prepared at a molar ratio of 20 for 1 h, followed by staining with Hoechst 33342. GFP and BFP channels disclosed cellular locations of QDs and nuclei, respectively. Bright-field images were obtained to represent cell morphologies using a Motic AE31 fluorescent microscope with an enlargement of 200x. (**B**) A time course of cellular internalization of HL6/QD complexes. Cells were treated with serum-free medium, QDs, and HL6/QD complexes for 0–30 min. The fluorescent intensity was measured by flow cytometric analysis. Significant differences were determined when the *P*-value was less than 0.05 (*) or 0.01 (**). Data are shown as mean ± SD from four independent experiments performed in triplicates for each treatment group.

The QD delivery capabilities of HL6 CPPs were assessed using 20 molar HL6/QD complexes incubated with A549 cells, followed by staining with Hoechst 33342. Fluorescent microscopy detected green fluorescence in the cells treated with HL6/QD complexes, but not in control cells or in cells treated with QDs alone (Fig. 4A). Quantitative fluorescent intensity was determined after protein transduction, using cells treated with 20 molar HL6/QD complexes and then analyzed using flow cytometry. Cellular uptake of QDs mediated by HL6 started within 5 min and continued to show high protein transduction over time (Fig. 4B). These results indicate that HL6 CPPs are capable of quickly delivering QDs into cells.

### Molecular mechanism of cellular uptake of HL6/QD complexes

**Figure 5:**
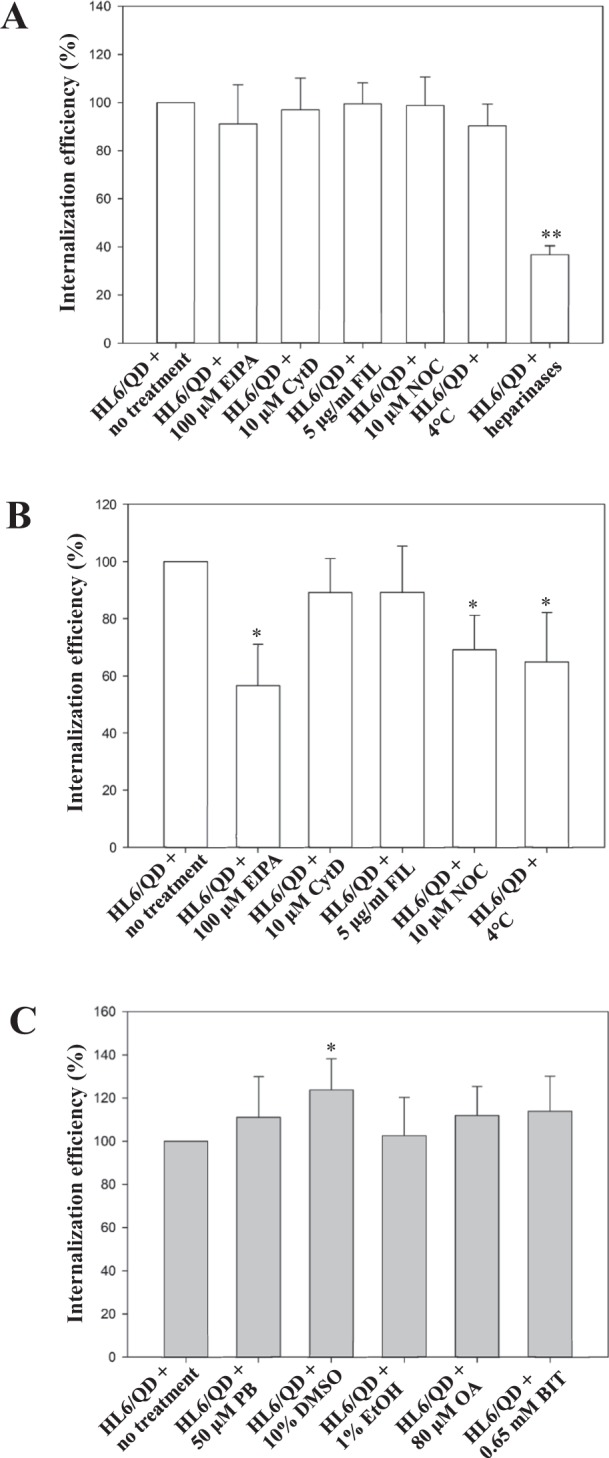
Mechanistic studies of cellular uptake of HL6/QD complexes. (**A**) Effect of endocytic modulators on cellular internalization of HL6/QD complexes after 5 min. HL6/QD complexes (prepared at a ratio of 20 at 37 °C for 1 h) were used to treat A549 cells in the presence or absence of pharmacological inhibitors (including EIPA, CytD, FIL, and NCO), low temperature treatment (4 °C), and heparinases for 5 min. Flow cytometry was used to analyze the cells after treatment for 5 min. Significant differences were determined when the *P*-value was less than 0.01 (**). Data are shown as mean ± SD from twelve independent experiments for each treatment group. (**B**) Effect of endocytic modulators on cellular internalization of HL6/QD complexes for 30 min. HL6/QD complexes (prepared at a ratio of 20 at 37 °C for 1 h) were used to treat A549 cells in the presence or absence of pharmacological inhibitors (including EIPA, CytD, FIL, and NCO) and low temperature treatment (4 °C) for 30 min. Flow cytometry was used to analyze the cells after treatment for 30 min. Significant differences were determined when the *P*-value was less than 0.05 (*). Data are shown as mean ± SD from eight independent experiments for each treatment group. (**C**) Effect of chemical enhancers of membrane transduction on HL6-mediated cellular uptake of QDs. Flow cytometry was used to analyze the cells treated with HL6/QDs complexes in the presence or absence of chemical enhancers PB, DMSO, EtOH, OA, or BIT. Significant differences were determined when the *P*-value was less than 0.05 (*). Data are shown as mean ± SD from eight independent experiments for each treatment group.

To reveal the mechanism of HL6-mediated cellular delivery of QDs, a series of inhibition studies were conducted. Flow cytometry was used to analyze cells treated by the HL6/QD complexes and pharmacological modulators, low temperature, or heparinases. Internalization of HL6/QD complexes was not affected by treatments with various endocytic inhibitors, including EIPA, CytD, FIL, NCO, and incubation at 4 °C for 5 min (Fig. 5A). In contrast, the transduction efficiency of HL6/QD complexes was dramatically decreased by treatment with heparinases. According to these findings, HL6/QD complexes are mainly delivered into cells using direct membrane translocation.

The transduction efficiency of HL6/QD complexes was decreased by treatment with pharmacological modulators EIPA, CytD, or NCO at 37 °C for 30 min, or incubated at 4 °C for 30 min (Fig. 5B). This suggests that macropinocytosis and clathrin-dependent endocytosis become involved in cellular uptake of HL6/QD complexes after extended periods of exposure. Together, these results indicate that direct membrane translocation is initially evident as the main uptake mechanism for HL6/QD complexes during cellular internalization. Subsequently, endocytosis contributes to the cellular uptake of HL6/QD complexes.

The HL6/QDs complex direct translocation was evaluated using several chemical enhancers of membrane transduction and analyzed using flow cytometry. Cells were treated with HL6/QDs complexes in PB, DMSO, EtOH, OA, or BIT. Only DMSO treatment among all tested chemical enhancers had a significant positive effect on cellular internalization of HL6/QD complexes (Fig. 5C).

### Cytotoxicity of HL6-mediated cellular uptake of QDs

**Figure 6:**
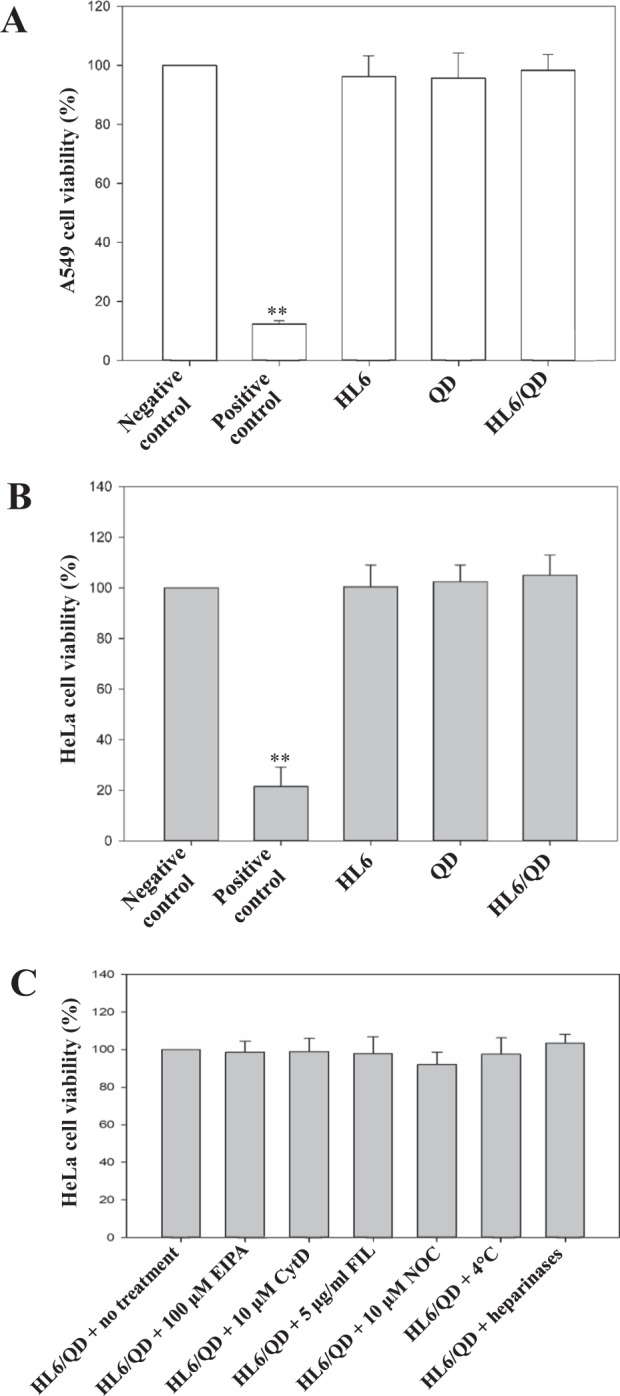
Cytotoxicity of HL6, QDs, and HL6/QD complexes in (**A**) A549 and (**B**) HeLa cells. Cells were treated with HL6, QDs, or HL6/QD complexes at 37 °C for 30 min. One hundred % DMSO and serum-free medium were used to treat cells at 37 °C for 30 min as positive and negative controls, respectively. (**C**) Cytotoxicity of HL6/QD complexes in the presence of a series of endocytic inhibitors in HeLa cells. Cells were treated with HL6/QD complexes (prepared at a ratio of 20 at 37 °C for 1 h) in the absence or presence of endocytic inhibitors, including EIPA, CytD, FIL, NCO (DMSO as the solvent), and heparinases (pretreated with heparinases I, II, and III for 6 h) at 37 °C for 30 min, or incubation at 4 °C (pretreated at 4 °C for 30 min) for 30 min. Cells were washed with PBS after treatment and then grown in complete culture medium at 37 °C for 24 h. The MTT assay was applied to assess cell viability. Significant differences compared with the negative control were determined when the *P*-value was less than 0.01 (**). Data are shown as mean ± SD from twelve independent experiments for each treatment group.

Cell viability after HL6-directed QD delivery was analyzed using the MTT assay. Cells were treated with HL6 alone, QDs alone, or HL6/QD complexes for 30 min, washed with PBS, incubated in complete culture medium at 37 °C for 24 h, and then analyzed using the MTT assay. No cytotoxicity was detected in A549 (Fig. 6A) or HeLa cells (Fig. 6B) for any of the treatments. Additionally, HeLa cells were not damaged by HL6/QD complexes in the presence of a series of endocytic inhibitors, low temperature, or heparinases (Fig. 6C).

## Discussion

This study demonstrates the novel HL6 peptides, which have flanking penta-histidine and mono-cysteine residues on the well-characterized cell-penetrating peptide L6, are able to deliver noncovalently complexed QDs into cells by direct membrane translocation. Cell viability assay with both A549 and HeLa cells indicates that none of the protein transduction components (HL6, QDs, and HL6/QD complexes) used in this study are cytotoxic. In our previous report, we demonstrated that L6/QD complexes enter cells by endocytosis^18^. Collectively, these studies demonstrate that the addition of polyhistidine peptides to an endocytic CPP allows internalization by direct membrane translocation. It is likely that HL6/QD complexes can enter cells by both direct membrane translocation and endocytosis simultaneously.

Endocytosis and direct membrane translocation contribute to cellular internalization of CPPs^2,6,15,29,30^. Recently, Futaki’s team has demonstrated that CPP concentration can play an important role in determining the mode of cellular internalization^15^. In general, the majority of studies investigating cellular uptake mechanisms of CPPs have utilized CPP concentrations ranging from 0.5 to 10 μM^31^. Direct membrane translocation mediated by CPPs tends to occur at relatively high concentrations of CPPs, while endocytosis is a dominant pathway at lower CPP concentrations. For instance, endocytosis is the uptake mechanism of 2 μM of fluorescently labeled R8 in leukemia cells^31^, while direct membrane translocation occurs at concentrations above 5 μM^15,31^.

GAGs are biopolymers with greatly negative charges across membranes of all animal cells. GAGs contain heparan sulfate and chondroitin sulfate, and are long polysaccharides made of alternating disaccharide units^32^. Heparinase acts on the extracellular surface to degrade GAGs but keeps the integrity of the plasma membrane intact^33^. The detailed molecular actions of GAGs, especially heparan sulfate proteoglycans, in cellular internalization of CPPs are still a matter of controversy^33–35^. It was previously postulated that direct membrane translocation originates from spatially restricted NZ regions on plasma membranes, with heparan sulfates on cell surface being necessary for internalization at these NZ sites^16^. Recently, loosening of lipid packing in the membrane bilayer was proposed to be the key factor governing the membrane translocation of CPPs^36^. Arginine-rich CPPs are able to interact with negatively charged constituents of plasma membranes, such as heparan sulfate proteoglycans and glycosylated lipids^15,36^. This interaction may induce dynamic alternations in membrane structure, including particle formation, local membrane inversion and pore formation, resulting in direct membrane translocation of CPPs into cells^13,15,36^. As revealed by flow cytometry, the uptake of HL6/QD complexes was reduced by 63.2% following heparinase digestion (Fig. 5A). These results are consistent with previous reports that enzymatic digestion by heparinases led to great suppression of direct membrane translocation of arginine-rich CPPs^15,16^.

The positive charges of cationic CPPs, most rich in arginines or lysines, are an important functional characteristic required for cell entry^2,37^. Surprisingly, polyhistidine peptides, such as a hexadeca-histidine (H16) peptide, were recently reported as novel CPPs in human HepG2 and other cells^37^ as well as in plant and yeast cells^38^. Cellular uptake of H16 peptides is mainly the result of macropinocytosis, and most H16 peptides localize in lysosomes and the Golgi apparatus^37^. Endosomolytic agents, such as hemagglutinin-2 (HA2)^39^, have been engaged to conquer endosomal entrapment and to enhance protein transduction efficiency. A modified PTD of transactivator of transcription (Tat) containing additional polyhistidine and cysteine residues^40^ and LAH4 peptides containing four histidines^41^ possessed endosomolytic properties. Similarly, the modified LKH-stEK CPP derived from the replacement of Lys residues of stEK CPP with His moieties was found to facilitate endosomal escape of short interfering RNAs (siRNAs) and promote >90% gene silencing^42^. Recently, polyhistidine-arginine (H6R6) peptide was demonstrated to show higher cell-penetrating efficiency and greater endosomal escape capacity compared to unmodified chitosan nanoparticles *in vitro*^43^. It has commonly been assumed that electrostatic interactions shared by both anionic cell membrane surfaces and cationic CPPs play a vital part in cellular internalization^15^. It should be noted that histidine possesses an imidazole ring with a pKa around 6.5, and thus is neutral without electric charge under physiological conditions. Nevertheless, the imidazole ring of histidine starts to be protonated in acidic tumor microenvironments^44,45^. Therefore, possible protonation of polyhistidine residues in acidic tumor microenvironments may facilitate cellular internalization of histidine-rich HL6. Additionally, we previously found that the nona-arginine CPP modified with additional polyhistidine and cysteine residues (becoming HR9) entered cells by direct membrane translocation, while the unmodified nona-arginine CPP entered cells by endocytosis^12^. Consistently with the previous study, HL6 with additional polyhistidine residues was shown to internalize into cells by direct membrane translocation, whereas the unmodified L6 CPP entered cells by endocytosis^18^. Collectively, our results indicate that polyhistidine peptides can play a determinant role in the mechanism of CPP-mediated protein transduction.

## Conclusion

A novel HL6 (CHHHHHRRWQWRHHHHHC) CPP containing polyhistidine peptides flanking the L6 CPP can generate stable complexes with QDs at an optimal molar ratio of 20 and then deliver QDs into cells. Mechanistic studies revealed that direct membrane translocation is a principal route of internalization for HL6/QD complexes. Cytotoxicity studies proved HL6, QDs, and HL6/QD complexes were not harmful to A549 and HeLa cells. Overall, HL6 CPP may be an efficient and biocompatible carrier of nanoparticles or therapeutic cargos in biomedical applications and theranostics.

## Supplementary information

Supplementary Figure S1
