# Supplementary material for: Polyhistidine facilitates direct membrane translocation of cell-penetrating peptides into cells
Source: Sci Rep. 2019 Jun 28;9:9398. doi: 10.1038/s41598-019-45830-8 (PMC6599048; doi:10.1038/s41598-019-45830-8)
Supplement: Supplementary file 1 — Supplementary Figure S1 [file 41598_2019_45830_MOESM1_ESM.pdf]

Polyhistidine facilitates direct membrane translocation of cell-penetrating peptides into cells

Han-Jung Lee, Yue-Wern Huang, Shiow-Her Chiou & Robert S. Aronstam

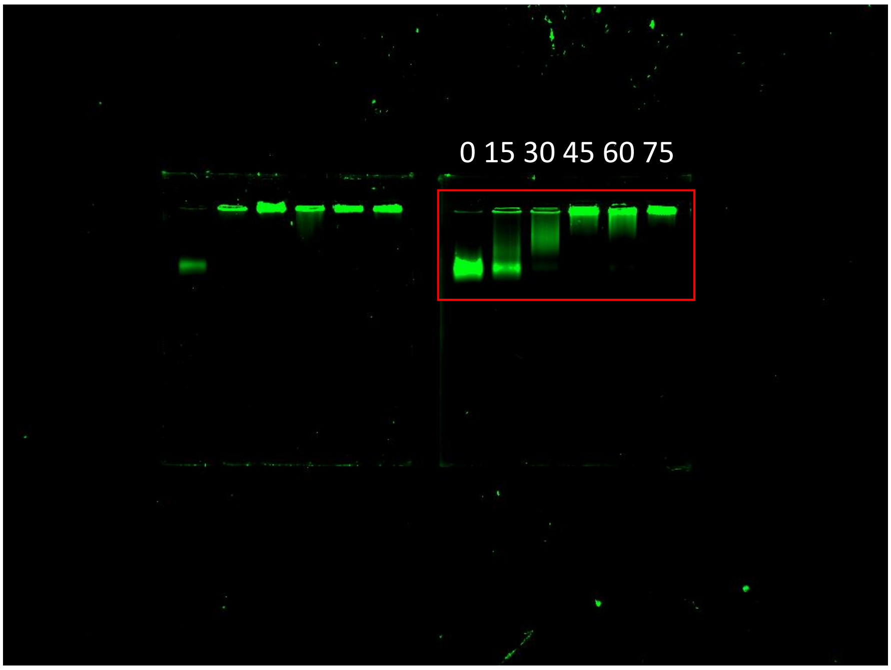

Supplementary Figure S1. A full-length gel of Figure 3A. Gel retardation assay showed that various amounts of HL6 were incubated with QDs at molar ratios of 0, 15, 30, 45, 60, and 75. Red line indicates the cropped representative image used in the article.
